# Peer-Led Adjunctive Interventions for Increasing the Reach of HIV Prevention and Care Interventions to Latino/x/e Men Who Have Sex with Men: a Scoping Review

**DOI:** 10.1007/s11904-024-00719-8

**Published:** 2025-01-07

**Authors:** Jahn Jaramillo, Jennifer V. Chavez, Michaela E. Larson, Audrey Harkness

**Affiliations:** 1 Department of Public Health Sciences, University of Miami Miller School of Medicine, Miami, FL USA; 2 Robert Stempel College of Public Health and Social Work, Florida International University, Miami, FL USA; 3 School of Nursing and Health Studies, University of Miami, 5030 Brunson Dr, Coral Gables, Miami, FL 33146 USA

**Keywords:** Scoping review, HIV prevention, HIV care, Latino men who have sex with men, Peer-led interventions, Adjunctive interventions

## Abstract

**Purpose of Review:**

Latino/x/e men who have sex with men (LMSM) in the United States are disproportionately affected by HIV. Peer-led adjunctive interventions show promise for enhancing engagement in HIV prevention and care among LMSM, but their effectiveness and implementation remain underexplored. This scoping review aimed to map existing evidence on peer-led interventions, identify gaps, and inform future research for enhancing HIV prevention and care among LMSM.

**Recent Findings:**

We followed PRISMA-ScR guidelines, covering literature from 2011 to 2022, using Covidence for systematic screening and data extraction. Articles were categorized by intervention aspects like delivery methods, outcomes, translational phases, theory-informed approaches, and cultural adaptation levels. The search yielded 613 records, with 23 meeting eligibility criteria, including 17 unique interventions. Interventions were delivered individually (57%), in groups (30%), to couples (4%), and via public campaigns (4%). Outcomes included HIV testing uptake (74%), treatment linkage (39%), PrEP uptake (22%), and PEP uptake (4%). Translational phases included formative (22%), pilot (26%), efficacy (22%), and effectiveness (22%). Cultural adaptations were surface (22%) and deep (13%).

**Summary:**

Findings indicate diverse peer-led interventions for LMSM, though many are in early stages of development. Further research is needed to move these interventions along the translational pathway to enhance their public health impact.

## Introduction

Latino/x/e men who have sex with men^1^(LMSM) in the United States (US) are disproportionately affected by HIV. If rates persist, about 20%of LMSM will be diagnosed within their lifetime [1, 2]. Despite the availability of evidence−based biomedical HIV prevention and treatment interventions, including pre−exposure prophylaxis (PrEP), post−exposure prophylaxis (PEP) and antiretroviral treatment (ART), these interventions inadequately reach LMSM [3–8]. Strategies are needed to facilitate the successful reach of HIV prevention and care biomedical interventions to LMSM.

Barriers to the successful reach of biomedical HIV prevention and treatment interventions to LMSM include social and structural obstacles, such as limited English proficiency, cultural differences in the expression of health concerns, inadequate interpreter services, HIV-related and intersectional stigma, social isolation, fear of deportation, lack of documentation, and medical mistrust [9–11]. Specifically in the case of PrEP, LMSM’s timely and consistent access is hindered by financial, logistical, social, and provider-related barriers, which limit the potential of PrEP to reduce HIV transmission among LMSM [12, 13].

Adjunctive interventions are interventions that are recipient facing and helps the recipient to initiate or adhere to a clinical intervention, such as PrEP, PEP, or ART [14] For example, behavioral interventions that use peers to increase LMSM’s use of or adherence to PrEP, PEP, or ART would be considered a adjunctive intervention. Peers, when used in the context of these types of adjunctive interventions, have been promising. For example, the employment of peers in patient navigation programs has improved engagement, linkage to care, and uptake of antiretroviral treatment (ART) among individuals living with HIV [15–17]. Peer delivered adjunctive interventions may be particularly relevant to LMSM, given prior findings that relational factors can promote LMSM’s engagement in PrEP services and research [18–20]. Given the central role of relational factors and evidence from some studies that peer involvement in HIV interventions promote positive engagement outcomes, peer-led adjunctive interventions may hold promise for engaging and linking men to PrEP and other HIV prevention and care interventions [8, 21, 22].

Due to the disproportionate HIV burden and insufficient reach of biomedical HIV prevention and treatment interventions to LMSM, the *Ending the HIV Epidemic* plan for the U.S. specifically named the importance of enhancing the reach of these critical tools to LMSM [23, 24]. In fact, modeling studies have shown that the *EHE* goals cannot be achieved without addressing racial/ethnic disparities in the HIV epidemic [25, 26]. While there is limited research on culturally appropriate peer-driven interventions for LMSM [24, 27], a growing body of literature have identified peer-led interventions in systematic reviews assessing HIV testing, treatment, and PrEP outcomes among LMSM [7, 28]. Thus, there is a need for culturally appropriate adjunctive interventions to scale up and disseminate PrEP, PEP, and ART to LMSM [27, 29, 30].

The CDC Compendium of Evidence-Based Interventions and Best Practices for HIV Prevention lists a variety of adjunctive interventions that have been shown to improve the reach of PrEP and ART to men who have sex with men in general [31]. Some of these adjunctive interventions leverage peers [32, 33]. Yet, few of these adjunctive interventions listed as “best evidence” in the compendium are specifically for LMSM. In fact, it is unknown how many adjunctive interventions that leverage peers and are for LMSM exist [7, 28]. For example, there may be other peer-delivered adjunctive interventions for LMSM that have not yet made it into the compendium, but that are promising or simply earlier in their development. There is also limited information about the features of these existing adjunctive interventions for LMSM [7, 8, 34].

This scoping review aims to characterize current knowledge about peer-delivered strategies that have been used to scale up and disseminate HIV prevention and treatment services to LMSM. Specifically, by outlining the priority population, study location, description of the intervention, and the goal, method, outcomes, and deliverers of the peer-led intervention within the included articles, we provide a comprehensive snapshot of the current state of the field. Through this scoping review, we aimed to understand the extent to which existing strategies were evidence-based and the next scientific steps that are needed to move evidence-based strategies into community practice. Results from this scoping review will help identify gaps in the literature and help inform future interventions that may leverage peer-delivered strategies to improve engagement of LMSM in HIV prevention and care services.

## Methods

We conducted a scoping review to review adjunctive interventions for increasing the reach of HIV testing, treatment, and PrEP. This scoping review was conducted following the Preferred Reporting Items for Systematic reviews and Meta-Analyses extension for Scoping Reviews (PRISMA-ScR) guidelines [35]. The protocol for this review can be accessed from the authors, but the review was not registered. The authors used Covidence, an online workflow platform for primary screening and data extraction for systematic reviews to conduct this scoping review (Covidence systematic review software, Veritas Health Innovation, Melbourne, Australia, available at www.covidence.org).

### Criteria for Studies

#### Population

Given the scope of the review, eligible articles must have included at least one Latino MSM in the analysis, or a description of the peer-led adjunctive intervention intended for/targeting LMSM if no formal analysis was conducted. There were no exclusions based on HIV status.

#### Adjunctive Interventions

In this review, we use the term “adjunctive interventions” to refer to interventions that are intended to increase LMSM’s use or adherence to other clinical interventions (e.g., PrEP, ART). There have been some semantic inconsistencies in the literature, with some studies calling these “implementation strategies” and others calling them “interventions;” while this is not necessarily resolved in the field, we use the term “adjunctive interventions” in this scoping review [36]. We define adjunctive interventions as supplementary strategies (e.g., strategies to support engagement and uptake) that assist recipients (e.g., clients) in participating in and adhere to a health intervention (e.g., PrEP), thereby supporting but not directly determining health outcomes [36]. As such, even if a study called an adjunctive intervention just an intervention or an implementation strategy, but its purpose was to increase LMSM’s use or adherence to these interventions, it was considered eligible for our scoping review.

Peer-led adjunctive interventions facilitated by individuals who shared similar characteristics or experiences with LMSM were included in our review. These interventions are typically guided or delivered by peers to promote well-being and health-related behaviors. They encompass activities like peer navigation to facilitate access to HIV care, peer-led HIV prevention programs to promote healthier practices and encourage HIV/STI testing, as well as leveraging influential peers via media platforms such as mobile apps, websites, and social media. The adjunctive intervention had to be consumer-facing, meaning that it sought to create change within LMSM to increase uptake or adherence. We included multilevel interventions, as long as there was at least one component that was a peer-led adjunctive intervention. The intervention could be delivered in English, Spanish, or both. We did not exclude articles based on study location. Studies had to have at least one LMSM among the participants and could employ a combination of both tailored and non-tailored methods, provided that LMSM were adequately represented in the overall composition.

#### Comparison Group

We did not require there to be a comparison group in order to include the study in this scoping review.

#### Outcomes

Studies that assessed the following outcomes were eligible: PrEP uptake and/or adherence; HIV testing; PEP uptake and/or adherence; HIV treatment linkage, retention, or adherence. The articles did not necessarily have to collect or analyze the outcomes of interest, but they were required to discuss and demonstrate how the intervention aimed to impact those outcomes. These outcomes could be assessed either quantitatively or qualitatively. Additionally, if available, we collected information on implementation outcomes such as acceptability, appropriateness, cost, feasibility, fidelity from the perspective of both consumers and implementers, and the translational phase of the adjunctive intervention (ranging from planning/pilot to efficacy, effectiveness, implementation, and broad scale up).

### Search Strategy

Following our pre-established scoping review protocol, our search strategy was developed through an iterative process to return maximally inclusive results related to peer-led adjunctive interventions delivered to LMSM. We conducted our search in the following databases: PubMed, Embase, MEDLINE (Ovid), Web of Science, and PsycINFO. Search result references were downloaded from each site and imported into Covidence for title and abstract screening. The team searched for PDFs of the full texts through the reference manager (Zotero), directly from the databases, or through the University’s library (interlibrary loan).

### Data Collection

#### Selection of Studies

Studies were assessed for eligibility using title and abstract screening, followed by a full text screen prior to data extraction. Upon importing references to Covidence, duplicate articles were removed. Two independent coders then screened the titles and abstracts of the remaining studies for eligibility. If a study appeared to meet inclusion criteria, or the coders needed to read the full text to confirm eligibility, the coders proceeded to pass the study through the full text review. Once all titles and abstracts were screened, any disagreements (instances where one coder voted for an article to pass through to full text review, while the other coder did not) were reviewed by the two coders, and a third adjudicator to assess eligibility. At full text review, the two independent coders reviewed the full text to ensure eligibility. Like title and abstract screening, any disagreements on full text reviews were reviewed by the coders and adjudicator to assess eligibility. Full manuscripts were included when accessible, while abstracts and presentations were excluded due to insufficient information for data charting. In some cases, there were multiple published studies about the same adjunctive intervention. We included each of these studies as long as they individually met inclusion criteria above (e.g., if a pilot test of an article was published, and a subsequent efficacy trial, both were included in the scoping review since they yielded different types of evidence).

#### Data Extraction and Management

Data was extracted using an extraction template built in Covidence. Relevant data for extraction included study location, sample size, intervention characteristics including: (1) the outcomes of interest (PrEP uptake and adherence, PEP uptake and adherence, HIV testing, treatment linkage, treatment adherence and retention), (2) translational phase (formative [i.e., quantitative or qualitative studies that developed peer-led adjunctive interventions], pilot, efficacy, effectiveness, hybrid effectiveness-implementation, implementation, community-wide dissemination), (3) how the intervention was delivered (individual, couples, group, e-health, mass media), (4) theoretical basis for the intervention, (5) whether it was tailored to LMSM (e.g. surface structure–adaptations that match materials or messages to observable, superficial characteristics of the target population versus deep structure–adaptations that address core cultural values, or ethnic, cultural, historical, social or environmental factors that may influence specific health behaviors), (6) inclusion of community-based participatory research strategies or community input into adjunctive intervention development (e.g. community advisory board consultation, inclusion of community members as co-investigators, community initiated interventions) and (7) main findings from the study. The two independent coders independently extracted the relevant data for each of the full text articles that was included. The third adjudicator reviewed any disagreements and selected the extraction that was most relevant to the goals of the project.

## Results

### Search and Selection of Evidence

**Fig. 1:**
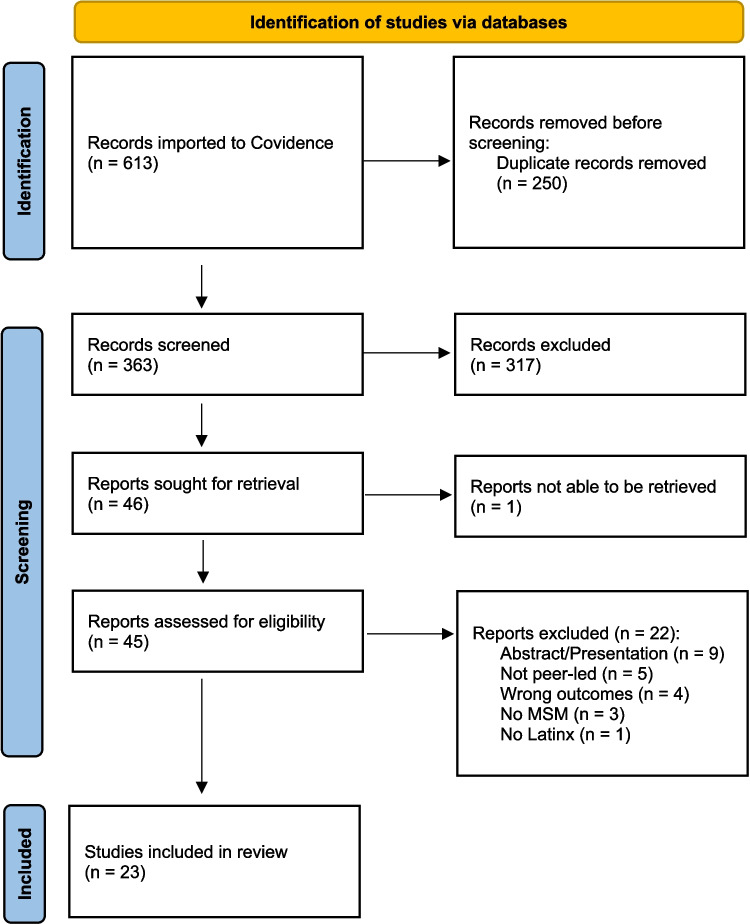
PRISMA flow diagram

Our search yielded a total of 613 articles. After removing duplicates, 363 studies remained. After title and abstract review and full report retrieval, 45 studies were selected to be fully characterized. Based on our eligibility criteria, we selected 23 studies to be included in our review (Table 1). Reasons for exclusion are listed in the PRISMA diagram (Fig. 1).

**Table 1:** Characteristics of accepted studies

Characteristic	*N* (%)
*Study location*	
United States	19 (83)
International (e.g., Brazil, Mexico)	4 (17)
Study population: age groups*	
Adolescents/youth (under 18 years)	1 (4)
Young adults (18–35 years)	15 (65)
Middle adults (36–50 years)	10 (44)
Older adults (> 50 years)	7 (30)
Not specified	8 (35)
*Study Population: Race/ethnicity*	
Latinx only	9 (39)
Latinx and other ethnic/racial groups	14 (61)
*Study population: immigration status*	
U.S. born only	1 (4)
Born outside of the U.S. only	2 (9)
Both	4 (17)
Not applicable (international studies)	4 (17)
Not specified	12 (52)
Outcomes targeted *	
HIV testing	17 (74)
PrEP uptake	5 (22)
PrEP adherence	3 (13)
PEP uptake	1 (4)
PEP adherence	1 (4)
HIV treatment linkage	9 (39)
HIV treatment adherence & retention	4 (17)
Translational phase	
Formative work	5 (22)
Pilot trial	6 (26)
Efficacy trial	5 (22)
Effectiveness trial	5 (22)
Hybrid effectiveness-implementation trial	2 (9)
*Intervention Theory Informed*	
Yes	13 (57)
No/Not specified	10 (43)
**Intervention Tailoring**	
Deep structure	4 (17)
Surface structure	5 (22)
Not specified	15 (65)
**Community Engaged/Informed**	
Yes	12 (52)
Not specified	11 (49)

*Note that these categories are not mutually exclusive, as the same intervention may have explored multiple ages and outcomes simultaneously

### Study Characteristics

Within the 23 studies that were included, there were a total of 17 unique adjunctive interventions to enhance the reach of evidence-based HIV prevention and treatment interventions to LMSM (Table 2). Only 9 studies focused exclusively on LMSM populations (36%). Three studies (13%) reported conducting major study activities in both English and Spanish, while 17% (*n* = 4) of studies conducted study activities exclusively in English, and 30% (*n* = 7) exclusively in Spanish. In terms of study location, 83% (*n* = 19) of studies were conducted in the United States. International studies of these adjunctive interventions were conducted in El Salvador (*n* = 1), Guatemala (*n* = 1), Brazil (*n* = 1) and Mexico (*n* = 1). Of the studies conducted in the US, 17% (*n* = 4) recruited both foreign-born and US-born individuals to participate. A vast majority (65%) of studies were conducted with young adults (18–35 years old), followed by middle adults (36–50 years old; *n* = 10, 44%) and older adults (greater than 50 years old; *n* = 7, 30%). Only 1 study focused on adolescents/youth populations (younger than 18 years old). The median sample size in these studies was 112 but ranged from 9 to 29,723. Of the 23 articles that were included, approximately 56% employed quantitative methods. The remaining 10 articles used a combination of qualitative analyses or mixed method approaches to evaluate peer led interventions. Intervention components are further described in Table 3.

**Table 2:** Studies included in scoping review

Citation	Study location	Translational phase	Study population	Outcomes assessed
Cunningham 2018	United States	Efficacy	Latino and Black/African American MSM and transgender women released from jail living with HIV	HIV Treatment Linkage; HIV Treatment Adherence & Retention
Davis 2018	Guatemala	Effectiveness	Latino MSM living with HIV	HIV Testing; HIV Treatment Linkage
Dickson-Gomez 2018	El Salvador	Effectiveness	Latino MSM, transgender women, and commercial sex workers	HIV Testing; HIV Treatment Linkage; HIV Treatment Adherence & Retention
Hurt 2022	United States	Pilot	Latino and Black MSM and transgender women who have sex with men diagnosed with HIV and/or syphilis	HIV Testing; HIV Treatment Linkage
Jackson 2022	United States	Pilot	Young Latino and Black MSM	PrEP Adherence
Jaganath 2012	United States	Formative	Latino and Black MSM	HIV Testing
Lightfoot 2018	United States	Hybrid effectiveness-implementation	Latino and Black MSM	HIV Testing
Molitor 2006	United States	Effectiveness	Latino, Black, and White MSM and non-MSM	HIV Testing; HIV Treatment Linkage
Pagkas-Bather 2020	United States	Formative	Latino and Black MSM	PrEP uptake; PrEP Adherence
Pascom 2016	Brazil	Pilot	Latino MSM, heterosexual men/women, and transgender persons	HIV Testing; HIV Treatment Linkage
Patel 2019	United States	Pilot	Young Latino and Black MSM	PrEP Uptake
Rhodes 2016	United States	Efficacy	Latino MSM (heterosexual but reported having sex with men)	HIV Testing
Rhodes 2020	United States	Efficacy	Latino MSM and transgender women	HIV Testing
Rocha-Jimenez 2021	Mexico	Pilot	Latino MSM, people who inject drugs, female sex workers, male sex workers, and transgender women	HIV Treatment Linkage; HIV Treatment Adherence & Retention
Shah 2021	United States	Effectiveness	Immigrant/foreign-born Latino men, women, and transgender persons who have sex with men and women	PrEP Uptake; HIV Testing
Sun 2015	United States	Formative	Gay, bisexual, and transgender persons	HIV Testing
Swendeman 2019	United States	Hybrid effectiveness-implementation	Gay, bisexual, and transgender persons	PrEP Uptake; PrEP Adherence; PEP Uptake; PEP Adherence; HIV Testing; HIV Treatment Linkage
Vissman 2009	United States	Formative	Latino non-MSM (reported providing services/support to other Latino MSM)	HIV Testing
Wohl 2011	United States	Pilot	Latino and Black MSM living with HIV	HIV Treatment Linkage; HIV Treatment Adherence & Retention
Young 2013	United States	Efficacy	Latino and Black MSM	HIV Testing
Young 2013	United States	Formative	Latino and Black MSM	HIV Testing
Young 2014	United States	Efficacy	Latino and Black MSM	HIV Testing
Young 2022	United States	Effectiveness	Latino and Black MSM	HIV Testing

**Table 3:** Peer-led adjunctive intervention components

Adjunctive intervention description/goal	Peer-led components	Location	Study type	*N* reported in study	Description of intervention recipients	Description of adjunctive intervention deliverers	Relevant outcomes assessed	Citation
**LINK LA**: A peer navigation intervention to support men and transgender women released from jail living with HIV through the continuum of HIV care to sustain viral suppression.	Trained peer navigators provided counseling, goal setting, problem-solving; accompanied participants to HIV care visits; facilitated communication with clinicians, access to services, and offered peer support.	California	Randomized Control Trial	Total: 356 Latino: 110 MSM: 201 LMSM: Not specified at this intersection	Latino, Black/African American, White MSM, non-MSM, and transgender women released from jail living with HIV	Peer navigators selected for experience relevant to post incarcerated persons (and not “matched” to participants based on demographic characteristics)	HIV Treatment Linkage; HIV Treatment Adherence & Retention	Cunningham 2018
A health navigation program for MSM living with HIV to support timely linkage to HIV care	Trained peers supported intervention activities, provided emotional support, built relationships, & assisted MSM in overcoming fear, stigma, & structural barriers to accessing HIV care.	Guatemala	Cross-sectional (Qualitative interviews)	Total: 19 LMSM	Latino MSM living with HIV	Universidad del Valle de Guatemala’s HIV Unit, peer educators, health navigators, and psychologist	HIV Testing; HIV Treatment Linkage	Davis 2018
A national HIV combination prevention intervention to decrease sexual risk behaviors, increase testing rates, & improve linkage to HIV treatment and adherence among MSM, commercial sex workers and transgender women	Trained peer outreach workers, provided prevention interventions, including risk identification, condom distribution, HIV testing encouragement, & linkage to care support.	El Salvador	Cross-sectional (Qualitative interviews)	Total: 78 health personnel LMSM: Not specified; study description indicates staff served LGBTQ + communities (including LMSM) & representative of populations they served.	Latino MSM, transgender women, and commercial sex workers	Clinic staff & peer outreach workers	HIV Testing; HIV Treatment Linkage; HIV Treatment Adherence & Retention	Dickson-Gomez 2018
**MATRix-NC**: Expansion of disease intervention specialist services to provide comprehensive care and support to Latino and Black MSM and transgender clients living with HIV and/or syphilis	Trained peers identified as “seeds” for chain-referral recruitment to reach individuals within their sociosexual network at risk of HIV & syphilis.	North Carolina	Cohort/Longitudinal	Total: 92 Latino: 15 Gay (lesbian or gay): 48 Male (cisgender): 66 LMSM: Not specified at this intersection	Latino and Black MSM and transgender women who have sex with men diagnosed with HIV and/or syphilis	Disease intervention specialists & peers	HIV Testing; HIV Treatment Linkage	Hurt 2022
**ESTEEM ConneCT**: Group intervention to improve mental health, reduce stigma-related stressors, enhance coping strategies, & increase sexual health, including PrEP use, among Latino, Black gay, bisexual, and other men who have sex with men	Peer-to-peer learning to promote healthy peer norms, role modeling, & social connections.	Connecticut	Cohort/Longitudinal	Total: 21 Latino: 7 Gay/bisexual/queer: 21 LMSM: Not specified at this intersection	Young Latino and Black MSM	Therapists selected based on CBT knowledge, group therapy experience, work with Latino/Black MSM, and understanding of intersectionality. Although the deliverers were professionally trained, they also had shared identities with the intervention recipients, including a Black queer man and Latinx gay man.	PrEP Adherence; PrEP Uptake (Note that the intervention also targeted mental health/substance use outcomes, but for the purpose of this scoping review the intervention was included because another targeted outcome was PrEP uptake/adherence).	Jackson 2022
**Project T**: A social network intervention to increase testing uptake & reduce undiagnosed HIV among Latino and African American MSM through HIV self-test kit distribution.	Peer recruiters distributed self-test kits to members of their social/sexual networks & provided support to those who tested positive in linking to care.	California	Cross-sectional	Total: 114 Latino: 38 Gay/bisexual: 97 LMSM: Not specified at this intersection	Latino and Black MSM	Latino/African American MSM and transgender women who have sex with men peer recruiters aged 18–45 who felt comfortable discussing HIV with a friend	HIV Testing	Lightfoot 2018
**California Bridge Project**: An intervention to locate and engage out-of-treatment individuals living with HIV and link them to HIV care and treatment services	Project staff called bridge workers served as peers and role models to enhance trust & engagement in care; were responsible for conducting ongoing outreach, providing immediate services or referrals for HIV counseling, testing, & assessing barriers to care.	California	Cross-sectional	Total: 325 Latino: 146 MSM: 158 LMSM: Not specified at this intersection	Latino, Black, and White MSM and non-MSM	Peer-based staff purposely hired with characteristics like those of the intended recipients of the intervention	HIV Testing; HIV Treatment Linkage	Molitor 2006
Peer navigation for PrEP use and its acceptability among Latino and Black MSM (formative)	Peer navigators to provide support & guidance in increasing PrEP access.	Washington	Cross-sectional	Total: 95 LMSM: 63	Latino and Black MSM	The purpose of this study was to develop the peer navigator intervention. Therefore, there were not intervention deliverers for the study. Rather, the study identified qualities that would make for an effective peer working with Latino and Black MSM to improve PrEP uptake & adherence. The study suggested peer navigators should be matched to participants based on sexual orientation, race, age, culture, & neighborhood.	PrEP Uptake; PrEP Adherence	Pagkas-Bather 2020
**Viva Melhor Sabendo*** (Live Better Knowing)*: A peer point-of-care testing intervention to expand HIV testing among key populations in Brazil	Trained peers administered oral fluid rapid tests & conducted HIV testing at social venues frequented by key populations, working in partnership with non-governmental organizations.	Brazil	Cross-sectional	Total: 29,723 LMSM: 6,055	Latino MSM, heterosexual men & women, transgender persons	Peers	HIV Testing; HIV Treatment Linkage	Pascom 2016
**Empowering With PrEP (E-PrEP)**: A peer-led social media-based intervention to facilitate PrEP use among young Latino and Black gay and bisexual MSM	Trained peers disseminated an online messaging campaign to their existing online networks, providing education about PrEP, increasing motivation to use PrEP & facilitating access to PrEP.	New York	Randomized Control Trial	Total: 162 LMSM: 29	Young Latinx and Black MSM	Peer leaders from the target population (young Black and Latino, gay, bisexual, and other MSM)	PrEP Uptake	Patel 2019
**HoMBReS*** (Hombres Manteniendo Bienestar y Relaciones Saludables; Men Maintaining Wellbeing and Healthy Relationships)*: A community-level lay health advisor HIV prevention intervention to promote consistent condom use and HIV/STD testing among heterosexual Latino men	Trained lay health advisors leveraged their peer relationships/social networks to disseminate HIV prevention messages, provide health education, and serve as community advocates.	North Carolina	Cross-sectional (Qualitative interviews)	Total: 9 LMSM: Heterosexual peers provided services to community including LMSM	Latino MSM and non-MSM	Navegantes (peer navigators) selected for personal characteristics (humor, self-esteem, dedication, respectfulness, and realism), performance characteristics (ability to communicate clearly, read low-literacy Spanish-language materials, collect data), and situational characteristics (having sufficient time and reliable transportation to fulfill their roles effectively).	HIV Testing	Vissman 2009
			Report/Review	LMSM: Report mentions intervention targeting Latino MSM				Rhodes 2016
**HOLA*** (Hombres Ofreciendo Liderazgo y Ayuda; Men Offering Leadership and Help)*: A peer navigation intervention to increase HIV testing and condom use among immigrant Spanish-speaking Latino gay, bisexual and other men who have sex with men and transgender women	Trained peers served as guides and advocates, providing support, information, and resources to promote HIV testing and condom use among their social networks.	North Carolina	Randomized Process Evaluation	Total: 11 LMSM: 10	Gay, bisexual, and transgender persons	Navegantes (peer navigators) selected based on leadership qualities, dedication, respectfulness, sense of humor as well as ability to provide advice on sensitive issues, communicate effectively, and work within their social networks, having available time and reliable transportation.	HIV Testing	Sun 2015
			Randomized Control Trial	Total: 166 LMSM: 148	Latino MSM and transgender women		HIV Testing	Rhodes 2020
**Conexiones Saludables** (Healthy Connections): A community-based peer navigator intervention to support engagement in HIV care in a in low resource settings among Latino populations	Trained peer navigators provided information and education about HIV transmission and treatment, linked participants to HIV care and ancillary services, provided emotional and instrumental support, and facilitated engagement in HIV treatment and adherence to antiretroviral therapy.	Mexico	Cross-sectional	Total: 44 LMSM: 22	Latino MSM, people who inject drugs, female sex workers, male sex workers, and transgender women	Peer navigators selected who have a deep understanding of the sociostructural barriers faced by key populations living with HIV in Tijuana, Mexico	HIV Treatment Linkage; HIV Treatment Adherence & Retention	Rocha-Jimenez 2021
**Sólo Se Vive Una Vez** (You Only Live Once): A social marketing campaign promoting HIV screening and prevention for immigrant Latinos	As part of an outreach team, bilingual community health workers provided HIV education and free testing at various community-based venues, aiming to reach those at highest risk of HIV acquisition.	Maryland	Cross-sectional	Total: 427 Gay/bisexual: 20 LMSM: Not specified at this intersection	Immigrant/foreign-born Latino men, women, and transgender persons who have sex with men and women	Promotores (Latinx outreach team composed of bilingual community health workers)	PrEP Uptake; HIV Testing	Shah 2021
Technology-based behavioral interventions to promote routine and repeat testing for HIV and sexually transmitted infections among youth	Peer coaching and online group peer support, where youth engage with their peers through private social media platforms.	California & Louisiana	Randomized Control Trial (Protocol)	Total: 1,500 planned that include LMSM in eligibility criteria	Latino and African American gay, bisexual, and transgender homeless youth	Near-peer coaches were bachelor’s-level paraprofessionals who closely matched the participant populations in terms of age, ethnicity, gender, and sexual identity.	PrEP Uptake; PrEP Adherence; PEP Uptake; PEP Adherence; HIV Testing; HIV Treatment Linkage	Swendeman 2019
A youth-focused psychosocial case management intervention to engage and retain young Latino and African American gay men of color in HIV care	Trained and supervised case managers delivered the intervention in a nonjudgmental and culturally appropriate manner, providing support and guidance to the participants throughout the intervention period.	California	Single-arm Trial	Total: 61 LMSM: 22	Latino and Black MSM living with HIV	Para-professional, bachelor-level peer case managers.	HIV Treatment Linkage; HIV Treatment Adherence & Retention	Wohl 2011
Project HOPE: An internet-based intervention harnessing online social networking sites (specifically Facebook), to scale HIV prevention interventions to Latino and Black MSM	Peer leaders with experience in social media & community outreach delivered culturally aware HIV prevention messages using social media platforms and acted as influential figures within their communities.	California	Cohort/Longitudinal	Total: Description only of curriculum and the method of evaluation for the intervention targeting Latino and Black MSM	Latino and Black MSM	Latino /African American MSM peer leaders selected for experience in using Facebook, being a popular opinion leader or community leader, and interest in using social networking for health education.	HIV Testing	Jaganath 2012
			Randomized Control Trial	Total = 112 LMSM = 67				Young 2013
			Cross-sectional	Total = 57 LMSM = 34				Young 2013
			Randomized Control Trial	Total: 112 LMSM: ~90% Latino/African American MSM; exact LMSM count not specified				Young 2014
			Randomized Control Trial	Total: 900 LMSM: 620				Young 2022

### Adjunctive Intervention Characteristics

#### Intervention Outcomes

Regarding the outcomes that the adjunctive interventions were targeting, approximately 17 (74%) of studies sought to increase HIV testing [37–53], 5 (22%) sought to increase PrEP uptake [43, 45, 54–56], 9 (39%) sought to increase HIV treatment linkage [16, 37–39, 41, 42, 45, 57], and only 1 sought to increase PEP uptake (4%) [45]. Although they are comprehensively presented in Table 2, we highlight a few adjunctive interventions in the context of their outcomes here. For example, Jaganath et al. [47] utilized Community Popular Opinion Leaders to effectively disseminate HIV prevention information, facilitating online access to home-based HIV testing kits among Latino and African American MSM. These testing kits were made available to all participants, demonstrating the study’s emphasis on the outcome of HIV testing. Of those that evaluated PrEP uptake as an outcome, Jackson et al. [55] conducted a pilot test of a group intervention to address intersectional stigma, mental health, and HIV risk among gay and bisexual men of color and explored whether the intervention led increased PrEP use/adoption among Latino and Black MSM. For linkage to HIV treatment, Cunningham et al. [16] tested the impact of the LINK LA (Linking Inmates to Care in Los Angeles) peer navigation intervention on viral suppression in released inmates living with HIV. This 12-session, 24-week intervention featured trained peer navigators who offered counseling focused on goal setting and problem-solving in the context of HIV care and medication adherence. A protocol paper by Swendeman et al. [45] outlines a randomized controlled trial to test the effectiveness of text-messaging, online peer support groups, and coaching strategies in optimizing the HIV prevention continuum for youth, including utilization of PEP (in addition to condoms and PrEP).

#### Evidence of Tailoring to LMSM

We examined two types of cultural tailoring: surface-level cultural tailoring (i.e., using culturally appropriate language, images, symbols, and examples to make the intervention more relatable to the target audience without necessarily altering its core content or principles) and deep-level cultural tailoring (i.e., modifying the intervention’s content, strategies, or delivery methods to align with the cultural context and values of the target population). Few studies (*n* = 4, 17%) reported conducting deep structure adaptations to tailor the intervention to LMSM [43, 44, 48, 49]. Sun et al. (2015) [44] evaluated a adjunctive intervention that had deep cultural tailoring for LMSM, evidenced by the fact that the intervention addressed: factors that influence health focusing on cultural expectations, cultural values, and reciprocal determinism; and what it is like to live with HIV as a Latino MSM. In another example of deep structure cultural tailoring, Rhodes et al. (2016) [49] trained peers (referred to as *navegantes*) to change health-compromising norms associated with the sociocultural environment (e.g., machismo, fatalism, homophobia and transphobia, and discrimination) and perceptions of Latino men. Finally, Shah et al. (2020) [43] developed a website with culturally sensitive video modules that LMSM could select based on their perceived barriers to HIV testing and/or HIV-related stigmatizing beliefs.

Although they were more common than deep cultural tailoring, surface-level cultural tailoring (*n* = 5, 22%) was also uncommon in the adjunctive interventions identified in this scoping review [41, 52, 55, 56, 58]. Surface-level tailoring included translating the intervention components to Spanish [58], incorporating general race-related stress and resiliency in intervention sessions [55], hiring bilingual peers [41], developing electronic materials including photos, videos, and pop-art that were representative of young Latino and Black MSM [56], and Facebook conversations related to the sociocultural barriers to HIV/AIDS prevention, such as related to race, religion, and community [52].

#### Translational Phase

The scoping review identified adjunctive interventions that varied across the entire translational spectrum (Table 2). Specifically, studies described adjunctive interventions that were in each of the following phases of evidence development: formative (*n* = 5, 22%) [44, 47, 50, 52, 54], pilot (*n* = 6, 26%) [39, 42, 55–58], efficacy (*n* = 5, 22%) [16, 46, 48, 49, 51], effectiveness (*n* = 4, 22%) [37, 38, 41, 53], hybrid effectiveness-implementation studies (*n* = 2, 9%) [40, 45] and implementation study (*n* = 1, 4%) [43]. Lightfoot et al.’s hybrid implementation-effectiveness trial [40] assessed a social network adjunctive intervention to distribute HIV self-test kits to Latino and Black MSM. They evaluated the effectiveness of the adjunctive intervention in terms of uptake of HIV testing, HIV test results, and linkage to care outcomes. Shah et al. [43] described the *Sólo Se Vive Una Vez* adjunctive intervention to increase HIV testing among Spanish-speaking Latino MSM and transgender persons. They detailed the adaptation, implementation, and reach of the social marketing campaign, which showed a positive impact on promoting HIV testing in the community. This study represents the later stages of translational research, concentrating on implementing a previously tested intervention in a new context while evaluating its reach and effectiveness.

#### Delivery Format

Adjunctive interventions identified in this scoping review were delivered to individuals (*n* = 13, 57%) [16, 37–46, 57, 58], couples (*n* = 1, 4%) [47], in group settings (*n* = 7, 30%) [48–53, 55], or through public campaigns (*n* = 1, 4%) [56]. Nine studies (39%) leveraged mass/social media to deliver their interventions, using popular social networking sites [43–47, 51–53, 56]. For example, in the HOPE (Harnessing Online Peer Education) program, peer leaders (i.e., for Latino and African American MSM) used social media platforms such as Facebook and Twitter to disseminate HIV prevention messages to their social network [46].

#### Community Engaged/Informed Interventions

Almost half (*n* = 10; 43%) of the studies mentioned employing the use of community engaged approaches to develop the adjunctive intervention [40, 42–45, 48–50, 56, 57]. Strategies comprised including community members in the development of the study protocol [40], including the community in an advisory or consultative capacity [44, 45, 48, 56, 57] such as through community advisory boards [44, 45], partnering with community members as co-investigators [42, 48, 50], and establishing formal CBPR partnerships [48, 50]. For example, HOLA: *Hombres Ofreciendo Liderazgo y Ayuda* (Men Offering Leadership and Help), which we considered a adjunctive intervention because it seeks to increase HIV testing among Spanish-speaking, less-acculturated Latino MSM, was developed through a CBPR partnership in North Carolina [48]. The CBPR partnership through which HOLA was developed had been established across the span of 10 years, with members of the partnership including lay community members, organization representatives, and health professionals/researchers from the university, working together to develop, implement, and evaluate the intervention [48]. As another example, HoMBReS: *Hombres Manteniendo Bienestar y Relaciones Saludables* (Men Maintaining Wellbeing and Healthy Relationships), a lay health advisor adjunctive intervention to increase HIV testing among recently arrived, non-English-speaking Latino men was also developed through a CBPR partnership comprised of local health and Latino-serving CBOs, religious organizations, an AIDS service organization, the local public health department, several academic institutions, and a soccer league. The CBPR partnership in the HoMBReS project was actively involved across all levels of intervention development, ensuring community engagement and input in the design, implementation, and evaluation of the intervention [50].

#### Theory-Informed Peer-Led Interventions

Over half (*n* = 13, 57%) of the studies described adjunctive interventions that were guided by a specific theoretical or conceptual framework [16, 37, 38, 40, 43, 46–49, 53, 55, 56, 58]. Adjunctive interventions were informed by social and behavioral theories, including social cognitive theory [16, 48, 49], social justice frameworks [37], the transtheoretical model [38, 43, 58], stigma-related stress models [55], the community popular opinion leader model [47], social network theory [40], the Information-Motivation-Behavioral Skills theoretical framework [43, 56], empowerment education [48, 49], social support [48], and diffusion of innovations theory [46, 47, 53, 56].

## Discussion

Our scoping review identified 23 studies which described 17 unique adjunctive interventions. Of these, only 7 had some level of tailoring specifically for LMSM participants. The limited number of adjunctive interventions that are tailored specifically to LMSM indicates a significant gap in the existing literature regarding peer-led adjunctive interventions that address the specific needs and challenges faced by LMSM. Although it is well known that peer-led approaches to HIV prevention and treatment are quite promising in general [15, 17, 28, 59] and the high relevance of relational factors for influencing LMSM’s engagement in HIV services, there are relatively few tailored adjunctive interventions leveraging this influential force for LMSM in particular [59, 60].

Within this scarcity of research on peer-led HIV strategies for LMSM, it is notable that many of the interventions included both Black and Latino MSM. Although Black and Latino MSM may experience some common barriers to obtaining HIV-prevention services (e.g., stigma, financial constraints, limited trust in providers) [61], Latino and Black MSM communities are not monolithic groups [62]. Existing literature underscores the significance of tailored interventions that account for the distinct needs and lived experiences within these broader categories [62–64]. Within Latino and Black MSM communities, there exist diverse subgroups with unique cultural, social, and behavioral characteristics, highlighting the need for interventions that address the specific challenges and realities of each subgroup [7]. In our review, we identified limited information concerning the language used for intervention delivery (English, Spanish, or both) and the immigration status of participants within the reviewed articles. Accurate assessment of intervention effectiveness for further minoritized subgroups of Latino MSM (e.g., undocumented LMSM), and factors based on language or nativity, relies on researchers documenting the specific subgroups represented in their studies. Without this kind of information, it is challenging to ascertain whether the interventions are equally effective for diverse subgroups of LMSM. It is essential to empirically assess effectiveness and extent of tailoring in interventions. Although there are existing culturally tailored interventions, including some in the early stages of development, the key area for future research is whether we should focus on creating new interventions, advance the research on existing ones, or pursue both strategies.

Peer-led adjunctive interventions have demonstrated promise in promoting HIV prevention and care among LMSM [48, 65]. However, there can be a significant delay (i.e., 17-year gap) between the development of interventions and their widespread implementation in community settings [66]. To achieve the goals of the Ending the HIV Epidemic (EHE) initiative, it is essential that peer-led interventions that do exist for LMSM are rapidly moved into the later stages of translational research, including dissemination, implementation, and scale-up [67]. Our scoping review highlights a greater issue within research that was articulated by Beidas et al. [68], regarding how psychosocial interventions have traditionally followed a linear translational pathway similar to biological clinical trials. In the article, authors highlighted that strict adherence to this pathway may hinder progress in the field [68]. They proposed several recommendations to enhance evaluation efforts in interventions including bypassing traditional translational steps and directly conducting effectiveness or pragmatic trials [68]. They also emphasized the importance of incorporating measures of both effectiveness and implementation from the outset of interventions [68]. In our review, we found that only a few studies specifically focused on implementation outcomes, which resulted in a limited understanding of their real-world application and their implementation in the community. By leveraging implementation science frameworks to monitor and assess HIV peer-led strategies, we can better understand the impact they have on the success of interventions and make comparisons with other HIV prevention approaches used among LMSM [69, 70]. By focusing on the rapid translation of effective interventions, we can help to close the gap between intervention development and community-level use and achieve the goals of the EHE initiative [24].

Experts in intervention development recommend adopting approaches that include the adaptation of interventions [71, 72] and community-based strategies [73] to accelerate the dissemination of interventions to communities. Developing new interventions can be resource-intensive, potentially exacerbating disparities in healthcare access [71, 74, 75]. Leveraging existing evidence-based interventions with community involvement could foster culturally grounded and equitable solutions [29, 76]. In our review, less than half of the adjunctive interventions used community-based approaches, with only 2 interventions incorporating community advisory boards. Community-based approaches like community advisory boards (CABs) play a pivotal role in engaging the community in the development, adaptation, and tailoring of interventions that facilitate the delivery of biomedical prevention methods to LMSM [73, 77]. These proactive approaches accelerate the development and implementation of peer-led adjunctive interventions for HIV prevention and care among LMSM, potentially reducing stigma [69, 78], promoting status-neutral approaches to HIV service engagement [76], and capitalizing on existing infrastructure [79] for rapid dissemination in community settings. More research is needed to systematically document community-informed strategies and their impact on intervention effectiveness and outcomes [74, 80] for LMSM.

As interventions for LMSM continue to be developed and tested, it is essential to integrate theoretical frameworks that specifically consider the cultural, social, and structural factors which contribute to HIV risk and poor health outcomes among this population [81, 82]. Theory-based peer-led HIV interventions, which draw upon established behavioral and social science theories as well as evidence-based practices, are recognized as more effective [28, 59, 83]. By incorporating these frameworks, interventions can be designed, implemented, and evaluated in a manner that maximizes their effectiveness for LMSM [84]. For example, theoretical frameworks like the Theory of Planned Behavior (TPB), could play a significant role in guiding the development of peer engagement, peer training, and peer influence strategies to cater to the specific needs and preferences of LMSM in HIV-related services. While several studies in our review mentioned the use of theories to guide intervention development, less than half of the studies did not include explicit details on the operationalization of these theories (e.g., such as their application in guiding peer training or informing the development of intervention manuals and topics). Future research on HIV peer-led interventions for LMSM could focus on integrating theoretical frameworks in a more comprehensive manner, elucidating how multiple theories can be synergistically employed, and specifying the specific elements employed across the intervention development process. These additional details can provide valuable guidance to researchers seeking to adapt, tailor, and implement peer-led interventions for LMSM within their own contexts.

### Limitations

While this review is one of the first to our knowledge to examine the state of the science on peer-led adjunctive interventions to increase the reach of HIV prevention and care for Latino MSM using a rigorous approach, the findings should be interpreted considering several limitations. First, a scoping review is used to examine the current state of knowledge of a particular topic without in-depth data synthesis or evaluation of study design, therefore, we did not formally evaluate effect sizes or study quality, limiting our ability to evaluate the strengths or weaknesses of the methodologies employed by the studies included. Second, although our search strategy was constructed iteratively, and in consultation with several subject matter experts, there is a possibility that we may have overlooked articles that would otherwise meet inclusion criteria. However, despite the aforementioned limitations, our scoping review exhibits several notable strengths. These strengths encompass a comprehensive examination of multiple databases without language or geographic restrictions, a discussion of implementation strategies, the inclusion of interventions spanning different stages of the translational pipeline, and the identification of several areas for future exploration in relation to this unique population.

## Conclusion

In conclusion, this scoping review focused on peer-led strategies for HIV prevention and care for Latino MSM. Through our comprehensive search, we identified a total of 23 articles that met the inclusion criteria. Our review highlighted key aspects of these interventions, including the delivery methods employed, intervention outcomes, translational phases, theory-informed approaches, evidence of tailoring to the unique needs of Latino MSM, and the degree of community engagement and input in the interventions. These findings shed light on the current landscape of peer-led strategies for HIV prevention and care for Latino MSM and emphasize the importance of tailoring and adapting interventions to meet the specific needs and experiences of sub-groups of LMSM. These insights offer valuable guidance for future research and the development of interventions that are responsive to the diverse needs of Latino MSM, ultimately contributing to improved health outcomes and health equity.

## Data Availability

No datasets were generated or analysed during the current study.

## Footnotes

1 In this paper, we use “Latino/x/e” as an inclusive and gender-neutral term to refer to individuals of Latin American descent. The term acknowledges and includes different gender identities within the Latino community. The “x” and “e” are used as placeholders to represent various gender-inclusive endings, allowing individuals to self-identify based on their gender preference.
