# Correction to: Peer-Led Adjunctive Interventions for Increasing the Reach of HIV Prevention and Care Interventions to Latino/x/e Men Who Have Sex with Men: a Scoping Review

**DOI:** 10.1007/s11904-025-00747-y

**Published:** 2025-06-23

**Authors:** Jahn Jaramillo, Jennifer V. Chavez, Michaela E. Larson, Audrey Harkness

**Affiliations:** 1 Department of Public Health Sciences, University of Miami Miller School of Medicine, Miami, FL USA; 2 Robert Stempel College of Public Health and Social Work, Florida International University, Miami, FL USA; 3 School of Nursing and Health Studies, University of Miami, 5030 Brunson Dr, Coral Gables, Miami, FL 33146 USA

**Correction to: Current HIV/AIDS Reports (2025) 22:12**

10.1007/s11904-024-00719-8

The original version of this article unfortunately contained a mistake.

The author has noticed a minor inconsistency in the Abstract. In the Recent Findings subsection, the sentence beginning “The search yielded 613 records...”, the number of included studies was incorrectly given as “22” but should have been “23”, as stated consistently in the main text.

The correct sentence should be written as follows:

The search yielded 613 records, with 23 meeting eligibility criteria, including 17 unique interventions. Interventions were delivered individually (57%), in groups (30%), to couples (4%), and via public campaigns (4%).

The original article has been corrected.

